# Pharmacokinetic study of omacetaxine mepesuccinate administered subcutaneously to patients with advanced solid and hematologic tumors

**DOI:** 10.1007/s00280-012-1963-2

**Published:** 2012-10-04

**Authors:** John Nemunaitis, Alain Mita, Joe Stephenson, Monica M. Mita, John Sarantopoulos, Swami Padmanabhan-Iyer, Nisha Nanda, Lyon Gleich, Annie-Claude Benichou, Adam Craig

**Affiliations:** 1 Mary Crowley Cancer Research Centers, 1700 Pacific Avenue, Suite 1100, Dallas, TX 75201 USA; 2 Cancer Therapy and Research Center, UT Health Center San Antonio Institute for Drug Development, San Antonio, TX USA; 3 Cancer Centers of the Carolinas, Greenville, SC USA; 4 ChemGenex Pharmaceuticals, Menlo Park, CA USA; 5 Medpace, Cincinnati, OH USA; 6 Stragen Services SAS, Lyon, France

**Keywords:** Omacetaxine mepesuccinate, Homoharringtonine, Cephalotaxine, Phase 1, Subcutaneous

## Abstract

**Purpose:**

Omacetaxine mepesuccinate is a first-in-class cephalotaxine demonstrating clinical activity in chronic myeloid leukemia. A subcutaneous (SC) formulation demonstrated efficacy and safety in phase 1/2 trials in patients previously treated with ≥1 tyrosine kinase inhibitor. This study assessed pharmacokinetics and safety of SC omacetaxine in patients with advanced cancers.

**Methods:**

Omacetaxine 1.25 mg/m^2^ SC was administered BID, days 1–14 every 28 days for 2 cycles, until disease progression or unacceptable toxicity. Blood and urine were collected to measure omacetaxine concentrations and inactive metabolites. Adverse events, including QT interval prolongation, were recorded. Tumor response was assessed at cycle 2 completion.

**Results:**

Pharmacokinetic parameters were estimated from cycle 1, day 1 data in 21 patients with solid tumors or hematologic malignancies and cycle 1, day 11 data in 10 patients. Omacetaxine was rapidly absorbed, with mean peak plasma concentrations observed within 1 h, and widely distributed, as evidenced by an apparent volume of distribution of 126.8 L/m^2^. Plasma concentration versus time data demonstrated biexponential decay; mean steady-state terminal half-life was 7 h. Concentrations of inactive metabolites 4′-DMHHT and cephalotaxine were approximately 10 % of omacetaxine and undetectable in most patients, respectively. Urinary excretion of unchanged omacetaxine accounted for <15 % of the dose. Grade 3/4 drug-related adverse events included thrombocytopenia (48 %) and neutropenia (33 %). Two grade 2 increases in QTc interval (>470 ms) were observed and were not correlated with omacetaxine plasma concentration. No objective responses were observed.

**Conclusions:**

Omacetaxine is well absorbed after SC administration. Therapeutic plasma concentrations were achieved with 1.25 mg/m^2^ BID, supporting clinical development of this dose and schedule.

## Introduction

Omacetaxine mepesuccinate is a first-in-class cephalotaxine in clinical development as an antileukemic therapy. Over 40 years ago, alcoholic extracts obtained from the bark of the evergreen plum yew *Cephalotaxus fortunei* were found to contain alkaloids with significant antitumor activity [1]. A semi-synthetic process was subsequently developed that utilized the leaves of the tree, rather than the bark, thereby allowing production of large quantities of highly purified omacetaxine, which is chemically identical to the natural product homoharringtonine [2].

Early phase 1 trials of omacetaxine in the United States in patients with a variety of solid and hematologic malignancies utilized short (over 60–90 min) intravenous (IV) infusions, and dose-limiting, life-threatening hypotension and tachycardia were observed at dose levels above 3–4 mg/m^2^ [3, 4]. Since then, further refinement of the omacetaxine dose and schedule via a low-dose, continuous IV infusion, or subcutaneous (SC) injection has been demonstrated to largely ameliorate these cardiovascular adverse effects [5–7].

Omacetaxine acts by binding to the A-site cleft of ribosomes and transiently inhibiting protein synthesis [8]. In vitro, omacetaxine induces apoptosis in leukemic cells due to a selective decrease in short-lived proteins, including the antiapoptotic proteins Mcl-1 and cMyc [9, 10]. In the mid-1990s, a phase 2 trial of omacetaxine in patients with chronic myeloid leukemia (CML) produced a complete hematologic response in >70 % of patients and major cytogenetic response in approximately 15 % [5]. These promising results were overshadowed by the introduction of imatinib, the first tyrosine kinase inhibitor (TKI) targeting the *BCR*-*ABL* oncogene in CML cells, and its approval in 2001 [11]. Although TKI therapy is now the standard of care for initial treatment of CML, interest in omacetaxine has been renewed in recent years with the recognition that resistance to initial TKI therapy occurs in approximately 25 % of patients [12–14] and that only 62 % of patients remain in complete cytogenetic remission at 6 years due to either acquired resistance or nonadherence [15]. Moreover, TKIs are not active against CML stem cells, promoting interest in other agents such as omacetaxine that may target leukemic stem cells [16].

The safety and efficacy of SC omacetaxine in patients with CML were evaluated in a phase 1/2, dose-escalation study [6]. In this study, SC omacetaxine was well tolerated up to a dose of 1.25 mg/m^2^ every 12 h [twice daily (BID)] for 14 days [5]. Subsequently, SC omacetaxine (at the same dose and schedule) demonstrated clinical activity and tolerability in two phase 2, open-label, multicenter studies in CML patients: one in patients with the T315I mutation who had failed prior imatinib [17] and the second in CML patients with resistance or intolerance to 2 TKIs [18]. Based on results of an analysis of these 2 studies [19], an application for United States Food and Drug Administration (FDA) approval of SC omacetaxine at this dose and schedule for patients with CML who failed previous treatment with 2 TKIs has been submitted.

In support of the clinical development of SC omacetaxine, the current study assessed the single- and multiple-dose pharmacokinetics and safety of SC omacetaxine mepesuccinate at a dose of 1.25 mg/m^2^ BID for 14 days every 28 days in patients with relapsed and/or refractory hematologic malignancies or advanced solid tumors.

## Methods

### Study design

This open-label, multicenter study was conducted in accordance with current FDA regulations, International Conference on Harmonisation Good Clinical Practice guidelines, the principles of the Declaration of Helsinki, and other applicable regulations and guidelines. Full ethical approval was granted by the institutional review boards at participating institutions. The study was registered at www.clinicaltrials.gov as NCT00675350.

### Patients

Adult patients with a diagnosis of relapsed or refractory CML, acute promyelocytic leukemia, acute myeloid leukemia (AML), or myelodysplastic syndrome (MDS), or those with advanced solid tumors who had exhausted or become intolerant to all available therapies, were eligible for participation. Additional inclusion criteria were life expectancy of >12 weeks, an Eastern Cooperative Oncology Group (ECOG) performance status ≤2, corrected QT interval <450 ms, and adequate organ function. Excluded were patients with previous omacetaxine treatment, NYHA Class III/IV heart disease, any uncontrolled cardiac condition, myocardial infarction within previous 12 weeks, solid tumors with known bone marrow or central nervous system involvement, active and uncontrolled systemic infection, chemotherapy within 4 weeks prior to study or radiation therapy within 6 weeks prior to study, or any medical or psychiatric condition rendering the patient unable to comply with study requirements. Written informed consent was obtained from all participants.

### Study drug administration

Omacetaxine 1.25 mg/m^2^ was administered SC every 12 h on days 1–14 of each 28-day cycle. The first dose was administered in the clinic by qualified site personnel who trained patients and/or caregivers in the proper technique for SC administration. Thereafter, the drug was given at home by the patient or caregiver who recorded each administration in a study diary; diaries were collected, and patient compliance was reviewed each week. The planned treatment duration was 2 cycles; if a response was documented after the first 2 cycles of treatment, patients were eligible for continued treatment.

The dosing schedule could be modified for adverse events (AEs). In patients who developed grade 4 neutropenia or grade ≥3 thrombocytopenia, treatment was delayed until recovery to grade ≤2, and the number of consecutive days of treatment was reduced by 2 days in subsequent cycles. For nonhematologic toxicity, treatment was delayed for grade ≥2 toxicity that was unresponsive to supportive care and considered possibly related to study drug. Upon resolution to baseline or grade ≤1, treatment was resumed at the same dose and schedule (for grade 2 events) or with a reduction in the number of consecutive dosing days for the remainder of that cycle only (for grade ≥3 events).

### Pharmacokinetic studies

Blood samples were collected from all patients on day 1 (predose and 0.5, 1, 2, 4, 8, and 12 h postdose); day 8 (predose); day 11 (predose and 0.5, 1, 2, 4, and 8 h after the 21st dose); day 15 (12–24 h after the 28th dose); and day 29 (predose). During study visits that included pharmacokinetic sampling, patients administered study drug under staff supervision. Urine for pharmacokinetic analysis was collected on days 1 and 11 (predose and 0–6, 6–12, and 12–24 h postdose).

Plasma and urine concentrations of omacetaxine and its 2 inactive metabolites, 4′-desmethylhomoharringtonine (4′-DMHHT) and cephalotaxine [20], were measured using a liquid chromatography–tandem mass spectrometry method developed, validated, and performed by Advion BioServices, Inc. (Ithaca, NY). Blood samples were collected in tubes containing dipotassium ethylenediamine tetraacetic acid and treated with 0.02 % paraoxon. For plasma analyses, 100 μL of plasma was processed by protein precipitation and analyzed using an XDB-Phenyl (2.1 × 50 mm, 5 μm) column at ambient temperature with the Sciex API 5000, Analyst Version 1.4.1, turbo ion spray, positive ionization, selected reaction monitoring detection system. Deuterated analogs of the compounds were used as internal standards. The lower limits of quantification of omacetaxine, 4′-DMHHT, and cephalotaxine in plasma and urine were each 0.100 ng/mL. The precision and accuracy of the method were acceptable, with a coefficient of variation percentage (CV%) ≤4.5 % for each analyte and bias values ranging from −5.2 to +5.3 %. For urinalysis, 400 μL samples of urine were processed using solid-phase extraction. Chromatographic conditions and equipment were identical to those involved in the plasma method. The precision and accuracy of the method were acceptable, with a CV% of ≤3.5 % for each analyte and bias values ranging from −7.3 to +4.0 %.

Noncompartmental pharmacokinetic analysis was conducted using WinNonlin^®^ Professional software, version 5.2.1 (Pharsight Corp, Mountain View, CA). C_max_ (days 1 and 11), T_max_ (days 1 and 11), and the minimum-observed plasma drug concentration during the steady-state dosing interval on day 11 (C_min_) were estimated from the plasma concentration versus time curve. The area under the plasma concentration versus time curve (AUC) from time 0 to the last sampling time point (AUC_last_; day 1 only) and AUC from time 0 to the end of a 24-h interval (AUCτ; day 11 only) were calculated using the linear trapezoidal rule. Terminal-phase elimination rate constant values were estimated by linear regression of the log concentration versus time profile, and used to calculate the terminal-phase half-life (t_1/2_). Derived pharmacokinetic parameters included (1) mean steady-state concentration, calculated as AUCτ/τ; (2) the AUC extrapolated to infinite time following the first dose (AUC_inf_); (3) the apparent clearance (CL/F) following the first dose and the dose on day 11, calculated as the dose divided by AUC_inf_ (day 1) or AUCτ (day 11); (4) the apparent volume of distribution in the terminal phase (V_z_/F); and (5) the mean accumulation ratio (R_acc_) between day 1 and day 11.

### Safety and efficacy assessments

At baseline, all patients underwent a complete physical examination and chest X-ray. Vital signs, ECOG performance status, complete blood count with differential and platelet count, and serum chemistries were evaluated at baseline and weekly during the study. Urinalysis was performed at baseline and on study days 11 and 29. A serial 12-lead electrocardiogram was conducted at baseline and on study days 1 (predose and 0.5, 1, 2, 4, 8, and 12 h postdose), 11 (following the 21st dose), 15, and 28. Corrected QT intervals [using Bazett’s correction and Fridericia’s correction of QT interval formulae (QTcB and QTcF)] were summarized with respect to change from baseline by visit and time point. At each visit, patients were monitored for AEs, and the duration, intensity, and causal relationship with study drug were evaluated. The severity of AEs was assessed using the National Cancer Institute’s Common Terminology Criteria for Adverse Events, version 3.0.

Baseline tumor evaluations were conducted within 28 days prior to first dose of study drug and repeated during the last week of cycle 2; these evaluations included bone marrow aspiration in patients with hematologic malignancies and computed tomography or magnetic resonance imaging in patients with solid tumors. In patients with solid tumors and measurable disease, response was assessed using RECIST criteria [21]. In patients with hematologic malignancies, response was assessed according to commonly accepted criteria for CML as well as the revised International Working Group criteria for AML and MDS [22–24].

## Results

### Patients and disposition

Twenty-one patients were enrolled at 3 United States sites from May to October of 2008. All patients received at least 1 dose of study drug. Thirteen patients (62 %) completed cycle 1 and went on to cycle 2; 10 of these patients (48 %) completed all 28 doses within 14 days in the first cycle. Two patients completed the second cycle of treatment, 1 of whom was approved to continue treatment and received a third cycle. Most common reasons for discontinuation were disease progression (52 %) and withdrawal of consent (24 %); no patients discontinued due to toxicity.

Patient characteristics are summarized in Table 1. Notably, patients exhibited a wide range in body weight (47.3–122.5 kg). Overall, a variety of tumor types were represented: 17 patients had solid tumors, including colon cancer (*n* = 6), pancreatic cancer (*n* = 3), lung cancer (*n* = 2), prostate cancer (*n* = 2), and squamous cell carcinoma, cervical cancer, hepatocellular carcinoma, and cancer of the parotid gland (*n* = 1 each). Hematologic cancers included AML, multiple myeloma, Hodgkin lymphoma, and diffuse large B-cell lymphoma in 1 patient each.

**Table 1:** Patient demographics and baseline characteristics

	All patients (*N* = 21)
Median (range) age, years	58 (40–76)
Male/female, *n* (percentage)	13 (62)/8 (38)
Race, *n* (percentage)	
Caucasian	21 (100)
ECOG PS, *n* (percentage)	
0	2 (10)
1	18 (86)
2	1 (5)
Median (range) weight, kg	71.4 (47.3–122.5)
Median (range) BSA, m^2^	1.8 (1.4–2.4)

*BSA* body surface area; *ECOG PS* Eastern Cooperative Oncology Group performance status

### Pharmacokinetic profile

Pharmacokinetic data were available for all 21 patients on day 1; 10 of 21 patients had complete data for estimating pharmacokinetic parameters on day 11. The pharmacokinetic parameters of omacetaxine are summarized in Table 2. Omacetaxine was rapidly absorbed into the blood following SC injection, as measurable plasma omacetaxine concentrations were observed at 0.5 h after the first dose in all but 1 of 21 patients. Mean T_max_ was 0.55 and 0.60 h on days 1 and 11, respectively. Mean C_max_ values were higher on day 11 (36.2 ng/mL) than on day 1 (25.1 ng/mL). AUC_inf_ (day 1) and AUCτ (day 11) values displayed a pattern similar to C_max_.

**Table 2:** Mean (CV%) pharmacokinetic parameters of omacetaxine 1.25 mg/m^2^ BID

PK parameter, unit^a^	Single dose (day 1; *n* = 21)	Multiple dose (day 11; *n* = 10)
C_max_, ng/mL	25.1 (56.0)	36.2 (55.6)
T_max_, h	0.55 (27.1)	0.60 (36.1)
C_min_, ng/mL	N/A	8.12 (91.1)
C_avg_, ng/mL	N/A	15.7 (72.3)
λ_z_, 1/h	0.111 (31.9)	0.109 (36.4)
t_½_, h	6.96 (35.0)	7.03 (31.8)
AUC_inf_, h ng/mL	136.2 (70.3)	N/A
AUC_last_, h ng/mL	91.7 (63.5)	N/A
AUC_τ_, h ng/mL	N/A	188.0 (72.3)
R_acc_	N/A	1.45 (16.2)
CL/F, L/h/m^2^	13.5 (64.0)	N/A
CL_ss_/F, L/h/m^2^	N/A	10.5 (76.3)
V_z_/F, L/m^2^	126.8 (63.9)	66.2 (59.2)

^**a**^In general, there was moderate to high interpatient variability, ranging from 30 to 75 % CV% in the key parameters, although the R_acc_ variability among the individual patients was lower, with a CV% of 16.2 %

λ_z_, terminal-phase elimination rate constant; AUC_τ_, AUC during a dosing interval, τ, at steady state; AUC, area under the plasma drug concentration versus time curve; AUC_inf_, AUC extrapolated to infinite time following the first dose; AUC_last_, AUC to the last sampling time; BID, twice daily; C_avg_, average steady-state plasma drug concentration; CL/F, apparent clearance divided by bioavailability (CL/F) values following the first dose and the dose on day 11; CL_ss_/F, apparent clearance divided by bioavailability (CL/F) values at steady state; C_max_, maximum-observed plasma drug concentration; C_min_, minimum-observed plasma drug concentration during the steady-state dosing interval on day 11; CV%, coefficient of variation (percentage); PK, pharmacokinetic; R_acc_, accumulation ratio between day 1 and day 11; t_1/2_, elimination half-life; T_max_, time of the C_max_; V_z_/F, apparent volume of distribution in the terminal phase

**Fig. 1:**
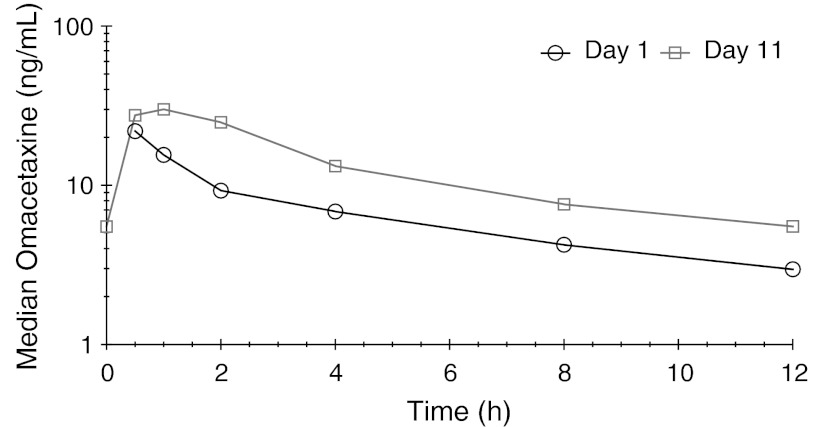
Median plasma omacetaxine concentration–time plots for days 1 and 11

Median omacetaxine plasma concentrations displayed a biexponential decay when plotted on a semilogarithmic scale (Fig. 1). The mean t_1/2_ after the first dose on day 1 (6.96 h) was nearly identical to that at steady state on day 11 (7.03 h). Mean apparent clearance (CL/F) on day 1 was comparable to that on day 11. There was some degree of omacetaxine accumulation during multiple twice-daily dosing, as indicated by a mean R_acc_ of 1.45. Measurable plasma concentrations were sustained over the dosing interval.

Notably, omacetaxine exposure was higher in males versus female patients, as determined by AUC_inf_ values estimated on days 1 and 11. This finding may be partially attributed to a lower body surface area observed in the female cohort, and as a result, females may have received a lower dose than males.

**Fig. 2:**
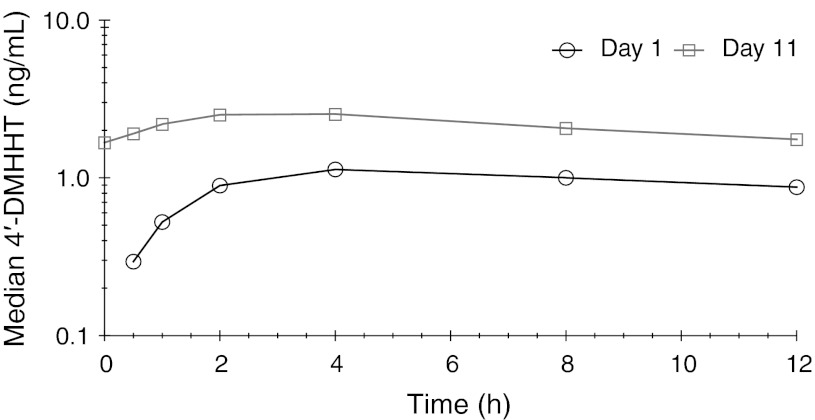
Median plasma 4′-DMHHT concentration–time plots for days 1 and 11. The *y*-axis scale is 1/10th that of Figure 1

Plasma concentrations of 4′-DMHHT were approximately 10 % of those for the parent compound; as observed with omacetaxine, median plasma concentrations were higher across the 12-hour sampling period on day 11 than on day 1. Peak plasma 4′-DMHHT concentrations were attained at approximately 3 h (day 11) to 5 h (day 1) after administration of omacetaxine. The elimination half-life of 4′-DMHHT was more than twice that of omacetaxine (approximately 16 h) and, in contrast to omacetaxine, the decay pattern for 4′-DMHHT was monoexponential (Fig. 2). Plasma concentrations of cephalotaxine were just above the lower limit of quantitation (0.100 ng/mL) in a limited number of samples in only 2 patients; these data were insufficient to permit calculation of pharmacokinetic parameters for this metabolite.

Urinary excretion of omacetaxine was relatively low, averaging 12 to 15 % of the administered dose on days 1 and 11, respectively. The amount of 4′-DMHHT excreted in the urine was 4 to 5 %, and the amount of cephalotaxine recovered in the urine was negligible at 0.07 to 0.14 %.

### Safety

The most common AEs (all grades) reported were anemia (71 %), thrombocytopenia (67 %), fatigue (62 %), and diarrhea (57 %) (Table 3). Seventeen patients experienced at least one grade 3/4 AE, most commonly thrombocytopenia (48 %), neutropenia (33 %), and anemia (19 %). Two patients experienced serious AEs that were considered by the investigator to be possibly or probably related to study drug (1 episode of grade 3 febrile neutropenia and 1 episode of grade 2 hypotension).

**Table 3:** Adverse events occurring in >10 % of patients

Event	All grades (*N* = 21) *n* (percentage)	Grades 3/4 (*N* = 21) *n* (percentage)
Hematologic		
Anemia	15 (71)	4 (19)
Thrombocytopenia	14 (67)	10 (48)
Neutropenia	9 (43)	7 (33)
Leukopenia	8 (33)	3 (14)
Nonhematologic		
Fatigue	13 (62)	2 (10)
Diarrhea	12 (57)	1 (5)
Anorexia	7 (33)	0
Vomiting	7 (33)	1 (5)
Nausea	6 (29)	1 (5)
Dehydration	6 (29)	0
Dyspnea	6 (29)	1 (5)
Injection-site erythema	5 (24)	0
Hypomagnesemia	5 (24)	1 (5)
Abdominal pain	4 (19)	0
Pain	4 (19)	2 (10)
Constipation	3 (14)	0
Hyponatremia	3 (14)	1 (5)

During cycle 1, the highest mean change in QTcB (6.2 ms) and QTcF (4.2 ms) occurred on day 1 at 8 h postdose. Two patients demonstrated a grade 2 QTcB >470 ms only on day 1 of treatment; 1 of these patients also showed a grade 2 QTcF >470 ms. Notably, the latter patient had a baseline QTcB of 463 ms. No apparent correlation was observed between peak plasma concentration (C_max_ on day 1 was 11.7 and 38.7 ng/mL) or steady-state concentration and absolute QTc value or change in QTc. No clinical events were documented in relation to QTc intervals >450 ms.

### Response

Seven patients underwent tumor evaluation at the end of cycle 2. No complete or partial responses were achieved. Stable disease was observed in 3 patients (1 each with prostate, cervical, and pancreatic cancer). Two additional patients (1 with squamous cell carcinoma and 1 with pancreatic cancer) underwent unscheduled tumor evaluations 6 to 7 weeks after treatment initiation which were consistent with radiographic evidence of stable disease. Objective responses were not observed in patients with hematologic malignancies.

## Discussion

This study evaluated the pharmacokinetic and safety profiles of omacetaxine administered SC at a dose of 1.25 mg/m^2^ BID for 14 days every 28 days in patients with advanced solid and hematologic tumors. Although formal studies have not been conducted to determine the bioavailability of omacetaxine, a cross-study comparison of systemic exposure following IV and SC administration indicates that bioavailability is approximately 70 to 90 % [25]. In the current study, SC omacetaxine was rapidly absorbed into plasma, as evidenced by mean t_max_ values of 0.55 and 0.60 h in single- and multiple-dose settings, respectively. Mean peak and steady-state omacetaxine concentrations at day 11 were approximately 1.5-fold higher than single-dose levels. In vitro studies in leukemic cells lines have shown the half-maximal inhibitory concentration (IC_50_) for omacetaxine to be 32 ng/mL or less [26]; notably, the mean C_max_ observed on day 11 was 36.2 ng/mL, indicating that the recommended dose and schedule may produce plasma concentrations associated with a pharmacodynamic effect. Drug exposure (AUC) was lower in females as compared to males, but the relationship to response is unclear. The relatively high apparent volume of distribution after SC administration suggests that omacetaxine is distributed beyond the vasculature into the tissues. Omacetaxine has previously been demonstrated to penetrate the blood–brain barrier [27].

The pharmacokinetic profile of the major metabolite of omacetaxine, 4′-DMHHT, was generally similar following single and multiple doses. Peak plasma concentrations of 4′-DMHHT, the primary metabolite of omacetaxine, were observed at 3 to 5 h after drug administration; the decline following maximal concentrations occurred more slowly than for omacetaxine, with a mean t_1/2_ of approximately 16 h, indicating slow conversion of omacetaxine to 4′-DMHHT and/or slow elimination of 4′-DMHHT. Steady-state AUC values showed that exposure to 4′-DMHHT was approximately 13 % of that for omacetaxine. In vitro evidence suggests that 4′-DMHHT has little or no pharmacodynamic activity [20]. Levels of cephalotaxine, the other known metabolite of omacetaxine, were undetectable in most patients.

Urinary excretion data indicate that less than 15 % of the administered dose of omacetaxine is excreted as unchanged drug, suggesting that dose adjustments may not be required in patients with renal impairment.

The toxicity profile of SC omacetaxine observed in this study was similar to that observed in other clinical studies [6, 17–19]. Myelosuppression was the major AE, primarily consisting of thrombocytopenia and neutropenia. Nonhematologic AEs were mainly grades 1 and 2 in severity. Omacetaxine produced no clinically apparent effects related to QT interval prolongation. Plasma concentrations of omacetaxine among patients with QTc intervals above 450 ms were within the range of those observed in patients with QTc values less than 450 ms.

In conclusion, the safety and pharmacokinetic profile of omacetaxine observed in this study, in particular the concentrations achieved and the t_1/2_, further support a twice-daily dosing schedule for omacetaxine as an effective alternative to continuous IV dosing.
